# Maternal Blood Adipokines and Their Association with Fetal Growth: A Meta-Analysis of the Current Literature

**DOI:** 10.3390/jcm13061667

**Published:** 2024-03-14

**Authors:** Ioakeim Sapantzoglou, Dimitrios-Efthymios Vlachos, Dimitrios Papageorgiou, Antonia Varthaliti, Kalliopi Rodolaki, Maria Anastasia Daskalaki, Alexandros Psarris, Vasilios Pergialiotis, Sofoklis Stavros, Georgios Daskalakis, Angeliki Papapanagiotou

**Affiliations:** 1 First Department of Obstetrics and Gynecology, “Alexandra” General Hospital, National and Kapodistrian University of Athens, 2-4, Lourou Str., 11527 Athens, Greece; kimsap1990@hotmail.com (I.S.); vlachos.dg@gmail.com (D.-E.V.); dimitris_papageorgiou@outlook.com (D.P.); antonia.varthaliti@hotmail.com (A.V.); kelli1_@hotmail.com (K.R.); psarris.alexandros@gmail.com (A.P.); pergialiotis@hotmail.com (V.P.); sfstavrou@yahoo.com (S.S.); 2 School of Medicine, European University of Cyprus, 6, Diogenous Str., Egkomi, Nicosia 2404, Cyprus; md181341@students.euc.ac.cy; 3 Department of Biological Chemistry, Medical School, National and Kapodistrian University of Athens, 75, Mikras Asias Str., Goudi, 11527 Athens, Greece; agpaoapanagiotou@gmail.com

**Keywords:** adipokines, fetal growth, growth restriction, leptin, adiponectin, visfatin, resistin, retinol-binding proterin-4

## Abstract

**Background**: Assessing fetal growth constitutes a fundamental aim within the realm of prenatal care. Impaired prenatal growth increases the risk of perinatal mortality, morbidity, and poor newborn outcomes. Growth restriction increases the risk of premature birth problems, as well as the risk of poor neurodevelopmental outcomes and future non-communicable disorders such as hypertension and metabolic syndrome as adults. The objective of this systematic review is to accumulate current literature evidence to assess the patterns of serum adipokine levels among women with growth-restricted fetuses and assess their potential alterations in those high-risk pregnancies. **Methods**: Medline, Scopus, CENTRAL, Clinicaltrials.gov, and Google Scholar databases were systematically searched from inception until 31 March 2023. All observational studies reporting serum adipokine values among women with appropriately grown and growth-restricted fetuses were held eligible. **Results**: The current systematic review encompassed a total of 20 studies, incorporating a patient population of 1850 individuals. Maternal blood leptin emerged as the adipokine most investigated, as evidenced by 13 studies encompassing a collective sample size of 1081 patients, all of which explored its potential correlation with intrauterine growth restriction. Elevated levels of leptin were detected in fetuses with intrauterine growth restriction, although the observed difference did not reach statistical significance. Furthermore, regarding adiponectin, the meta-analysis conducted indicated that there were not any statistically significant differences observed in the mean values of adiponectin. The available data on the remaining three adipokines were extremely limited, making it difficult for any solid conclusions to be extracted. **Conclusions**: Though limited and inconsistent, the existing data suggest that fetal growth restriction is not linked to leptin, adiponectin, visfatin, resistin, or RBP4. More substantial prospective studies are needed to comprehend the importance of established and novel adipokines.

## 1. Introduction

Fetal growth refers to the dynamic processes that take place during intrauterine life and lead to the development of a fetus. It occurs from the time of conception until birth and is typically measured in terms of gestational age. It is monitored by assessing ultrasound parameters, such as head circumference (HC), biparietal diameter (BPD), abdominal circumference (AC), and femur length (FL), that calculate the estimated fetal weight (EFW) and size for the specific gestational age (GA) [1].

The ideal birth weight ranges between 2500 and 4000 g for full-term infants. It is worth noting that there is a wide range of normal birth weights, and healthy infants can be born outside the reported range of values [2]. Small for GA (SGA) infants are defined as infants with a birth weight below the 10th percentile for the gestational age, who attain their genetic growth potential, in contrast with pregnancies complicated with fetal growth restriction (FGR), which are characterized by newborns who were unable to achieve their genetically determined potential, due to pathologic conditions.

Birth weight depends on various factors related to both maternal health and condition, as well as genetic and environmental factors. Especially, multiple pregnancies, placental insufficiency, premature birth, fetal growth restriction, and fetal factors such as chromosomal abnormalities are common entities that may affect fetal birth weight. Additionally, maternal factors such as size and weight, dietary habits, maternal genetic factors, as well as potential medical conditions, play a crucial role in determining the size and developmental dynamics of the fetus. Specifically, conditions such as gestational diabetes, hypertension, and preeclampsia can affect birth weight if not properly managed [3,4,5].

Lately, there has been particular interest in investigating the role of adipokines in birth weight and fetal development. Adipokines are proteins mainly produced by adipose tissue and play a significant role in the regulation of a wide range of processes, including metabolism, appetite, inflammation, angiogenesis, insulin sensitivity, and immune response. The levels of adipokines in maternal blood, and especially their influence on fetal growth, are an area of interest for contemporary researchers. Specifically, adipokines such as leptin, adiponectin, visfatin, resistin, and retinol binding protein (RBP)-4 are the focus of research by many scientists, exploring their relationship with fetal development and particularly with fetal growth restriction and, as a consequence, the birth of infants with low birth weight [6,7].

The main purpose of our systematic review is to determine a possible relationship between the levels of maternal blood leptin, adiponectin, resistin, visfatin, and RBP-4 and fetal growth, and the secondary purpose is to investigate the potential use of maternal blood adipokines as biomarkers for fetal growth abnormalities.

## 2. Materials and Methods

This systematic review and meta-analysis were designed according to the Preferred Reporting Items for Systematic Reviews and Meta-Analyses (PRISMA) guidelines. This review was registered in the PROSPERO international database for systematic reviews (reference: CRD42023428227).

### 2.1. Eligibility Criteria

The present systematic review included all observational studies (prospective/retrospective cohort, case-control, nested case-control, and cross-sectional) that reported serum levels of any adipokine (leptin, adiponectin, resistin, visfatin, and RBP-4) among pregnant women with and without fetal growth restriction. No trimester or laboratory assay restrictions were applied. Case-reports, small case series, letters to the editor, animal studies, and review articles were not included. Conference proceedings and abstracts were also planned to be excluded, as they lack important information that is necessary for the assessment of study limitations and quality of evidence. Uncontrolled studies, as well as studies measuring adipokines in amniotic fluid, umbilical cord, or placental tissue were excluded.

### 2.2. Information Sources

The literature search was primarily performed using the Medline, Scopus, Cochrane Central Register of Controlled Trials (CENTRAL), and Clinicaltrials.gov databases. Google Scholar, as well as the reference lists of the included studies, were also systematically searched in order to identify potential additional articles not recognized by the primary search. The date of the last search was set at 31 March 2023.

### 2.3. Search Strategy

The literature search was performed using the following terms: (“Adipokines” AND “fetal growth”), combined with a list of the examined adipokines. In particular, the main search algorithm was the following: (“Adipokines” OR adipocytokines OR leptin OR adiponectin OR resistin OR visfatin OR retinol binding protein-4 OR rbp-4) AND (“fetal growth” OR fetal growth restriction OR small for gestational age). No language or date restrictions were implemented during the literature search. The process of literature search was conducted by three authors independently (I.S., D.P., A.V.), while any potential disagreements were resolved through the consensus of all authors.

### 2.4. Study Selection

**Figure 1:**
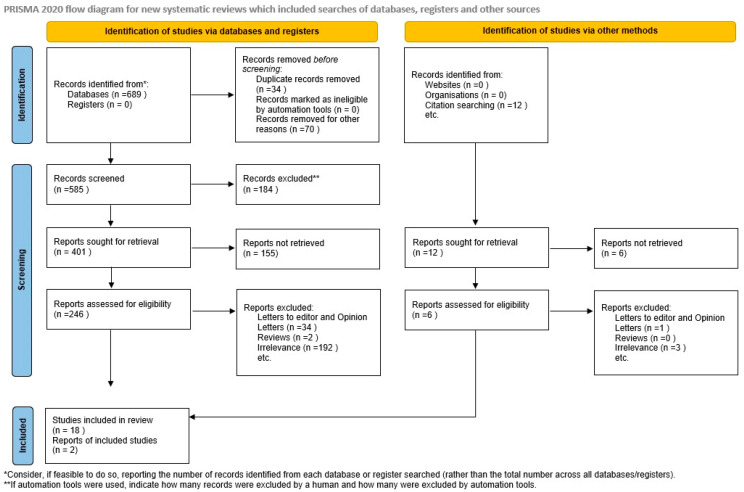
Search strategy.

The process of study selection was conducted in three consecutive stages. At first, the titles and abstracts of all electronic papers were screened to assess their potential eligibility. Subsequently, all articles that met or were presumed to meet the eligibility criteria were retrieved as full texts. Finally, all observational (both prospective and retrospective) studies reporting serum levels of the examined adipokines (leptin, adiponectin, resistin, visfatin, retinol binding protein-4) among women with fetuses affected by fetal growth restriction and healthy pregnant controls with appropriately grown fetuses were deemed eligible. Study selection was performed by two authors independently, while any potential discrepancies were resolved through their consensus (Figure 1).

### 2.5. Data Extraction

The following data were planned to be extracted from each included study: name of first author, year of publication, study design, inclusion and exclusion criteria, number of patients, maternal age, race, pre-pregnancy body mass index (BMI), BMI at birth, presence of diabetes mellitus, parity, smoking status, presence of Pregnancy Induced Hypertension (PIH) and preeclampsia (PET), caesarean section delivery rates, gestational age at delivery, birthweight, APGAR score, and umbilical cord Ph, as well as maternal serum levels of the evaluated adipokines. When important data were missing, attempts were made to contact corresponding authors. Data extraction was performed by three authors, while any possible disagreements were resolved through their consensus or by discussion with all authors.

### 2.6. Quality Assessment

**Figure 2:**
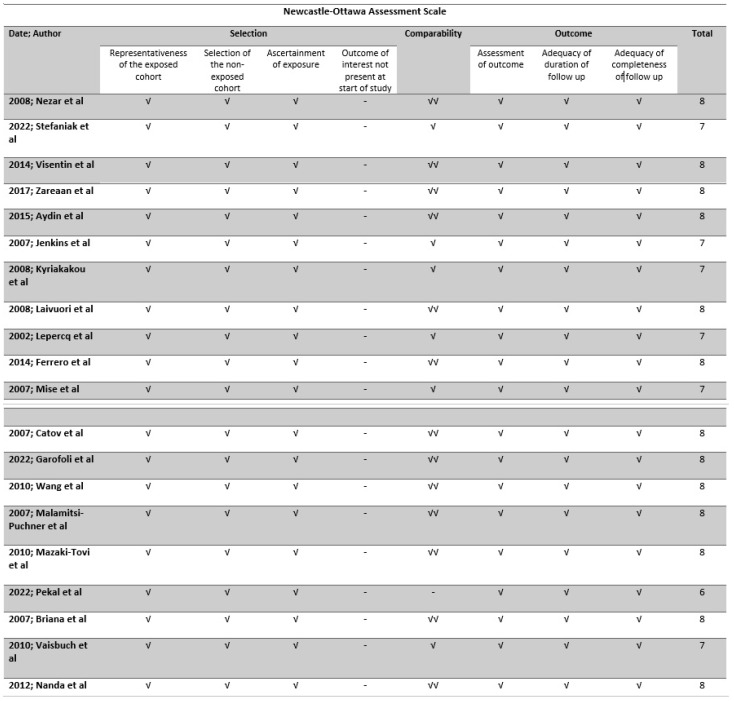
Newcastle–Ottawa scale (NOS) quality assessment of the included studies [9,10,11,12,13,14,15,16,17,18,19,20,21,22,23,24,25,26,27,28].√: the criterion under question is met, √√: two adjusting factors were taken into consideration.

The methodological quality of all included studies was evaluated using the Newcastle–Ottawa scale (NOS) tool, which is a widely utilized instrument for evaluating the methodological quality of non-randomized studies that are incorporated in systematic reviews and/or meta-analyses. The tool assesses each study based on eight criteria, which are divided into three categories: the selection of study groups (four criteria, and each of them may assign a maximum of one star), the comparability (one criterion that may assign a maximum of two stars) of the groups which was based on the maternal weight and the gestational age, and the ascertainment of either the exposure or outcome of interest for case-control or cohort studies (three criteria, and each of them may assign a maximum of one star), respectively. Stars are assigned to each quality item as a means of providing a rapid visual evaluation. Stars are allocated in a manner that grants the highest caliber studies a maximum of nine stars [8]. The tool was implemented by two authors independently, and any discrepancies were resolved through a third author. Overall, the risk of bias was assessed to be good, fair, or poor (Figure 2).

### 2.7. Data Synthesis

Statistical meta-analysis was performed with RStudio using the *meta* function (RStudio Team (2015). RStudio: Integrated Development for R. RStudio, Inc., Boston, MA URL http://www.rstudio.com/) in the R 4.3.2 environment. Statistical heterogeneity was not considered during the evaluation of the appropriate model (fixed effects or random effects) of statistical analysis as the considerable methodological heterogeneity (Table S1) did not permit the assumption of comparable effect sizes among studies included in the meta-analysis. Confidence intervals were set at 95%. We calculated pooled standardized mean differences (SMDs) as well as pooled 95% confidence intervals (CI) with the Hartung–Knapp–Sidik–Jonkman, instead of the traditional Dersimonian–Laird random effects model analysis (REM). The decision to proceed with this type of analysis was taken after considering recent reports that support its superiority compared to the Dersimonian–Laird model when comparing studies of varying sample sizes and between-study heterogeneity. Publication bias was considered using inspection of retrieved funnel plots for outcomes that included more than 10 studies, as well as with the Egger’s test which represents a linear regression analysis that takes into account the intervention effect estimates and their standard errors which are weighted by their inverse variance. Duval and Tweedies’ Trim n Fill test was then applied to evaluate the impact of a potentially unbiased meta-analysis and compare it to the findings of our study. The test firstly trims the studies responsible for funnel plot asymmetry and then, fills imputed missing studies (hypothetical in origin) using an assumption that supports a bias-corrected overall estimate.

The potential presence of small-study effects was evaluated with Rücker’s Limit Meta-Analysis and the possibility of p-hacking with inspection of the results of the p-curve analysis. 

### 2.8. Prediction Intervals

Prediction intervals (PI) were also calculated, using the *meta* function in RStudio, to evaluate the estimated effect that is expected to be seen by future studies in the field. The estimation of prediction intervals considers the inter-study variation in the results and expresses the existing heterogeneity at the same scale as the examined outcome. 

### 2.9. Trial Sequential Analysis

To evaluate the information size, we performed trial sequential analysis (TSA) in all meta-analyses that involved binary or continuous outcomes, which permits investigation of the type I error in the aggregated result of meta-analyses performed for primary outcomes that were predefined in the present meta-analysis. A minimum of 5 studies was considered as appropriate to perform the analysis. Repeated significance testing increases the risk of type I error in meta-analyses, and TSA has the ability to re-adjust the desired significance level by using the O’ Brien–Flemming a-spending function. Therefore, during TSA, sequential interim analyses were performed that permit investigation of the impact of each study on the overall findings of the meta-analysis. The risk for type I errors was set at 5% and for type II errors at 20%. Trial sequential analysis was not performed for pre-calculated effect size data, namely, hazards ratios, provided in this meta-analysis as currently, there is no algorithm available for this type of data. The TSA analysis was performed using the TSA v. 0.9.5.10 Beta software (http://www.ctu.dk/tsa/). 

## 3. Results

**Figure 3:**
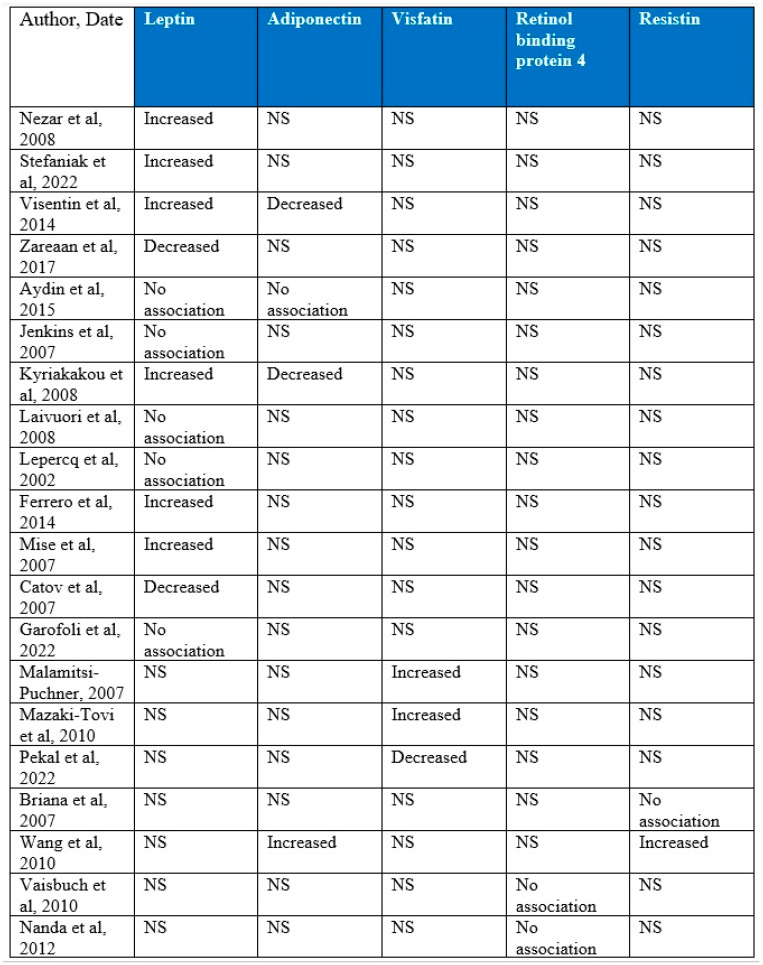
Summary of outcomes according to the adipokine investigated and the study included [9,10,11,12,13,14,15,16,17,18,19,20,21,22,23,24,25,26,27,28]. NS: not studied.

Overall, 20 studies were included in the present systematic review that included a total of 1850 patients. An overall summary of outcomes according to the adipokine investigated and the study included is presented in Figure 3. In terms of leptin, six studies revealed increased levels of the adipokine in pregnancies affected with growth-restricted fetuses compared to uncomplicated ones, two studies demonstrated decreased levels, and five studies revealed no association. In terms of adiponectin, decreased levels were demonstrated by two of the included studies, one study showed an increase in the levels of the adipokine, and one study concluded that no association could be revealed. Visfatin was investigated by three studies with two of them presenting increased levels in the affected cases, while one contradicted their results by demonstrating lower levels compared to the unaffected controls. No statistically significant differences were revealed between the affected and unaffected cases in terms of the levels of RBP-4 by the two studies that investigated a potential association. Resistin levels in growth-restricted fetuses were assessed by two studies with one revealing no differences while the other demonstrated increased levels in the affected pregnancies. 

The study design and the inclusion and exclusion criteria of the included studies that investigated leptin are depicted in Table S1, and the demographic characteristics of the patients are depicted in Table S2. The methodological characteristics of the included studies that investigated adiponectin are depicted in Table S3, and the demographic characteristics of the patients are depicted in Table S4. The most investigated adipokine was maternal serum leptin with 13 studies (one study involved two separate study groups that were taken into consideration individually) and a total of 1081 patients reporting its association with intrauterine growth restriction (IUGR). The methodological quality of included studies, assessed with the Newcastle–Ottawa scale score, revealed that most of the studies scored high, indicating that methodological bias was quite low. Selection bias might be partially present as the outcome of interest was already present during the conduct of included studies; however, in terms of comparability, the majority of included studies scored high, indicating that the IUGR and control groups were at least partially homogeneous in terms of the most prominent confounding factors (maternal weight and gestational, as already determined in the Section 2).

**Figure 4:**
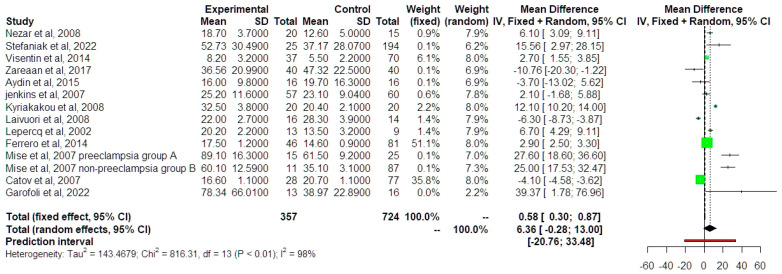
Μean differences of leptin between growth-restricted and appropriate-for-gestational-age newborns [9,10,11,12,13,14,15,17,23,24,25,26,27]. Forest plot analysis: Vertical line = “no difference” point between the two groups. Green squares = mean differences of individual studies; Diamond = pooled mean differences and 95% CI for all studies; Horizontal black lines = 95% CI; Horizontal red line = prediction intervals. Abbreviations: SD: Standard Deviation, CI: Confidence Intervals.

Leptin values were found to be increased in IUGR fetuses, although the actual result did not reach statistical significance (MD 7.88 ng/mL, 95% CI −0.03, 15.79, outcome from 13 studies, *p* = 0.0502) (Figure 4).

**Figure 5:**
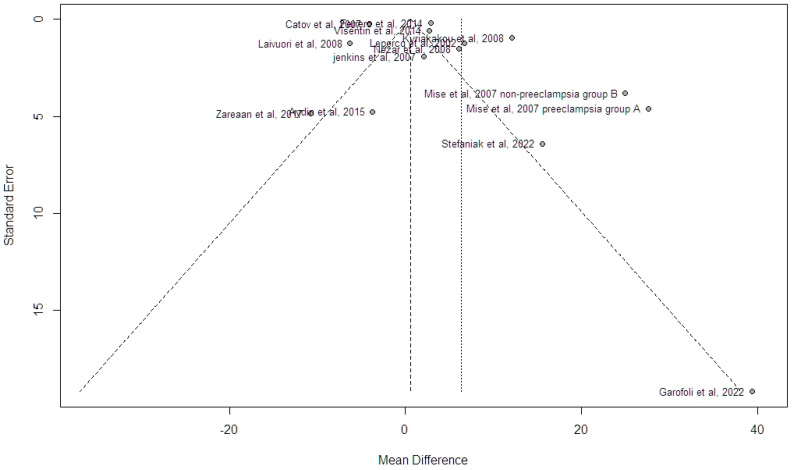
Funnel plot analysis indicates significant skewness in mean differences of included studies [9,10,11,12,13,14,15,17,23,24,25,26,27].

**Figure 6:**
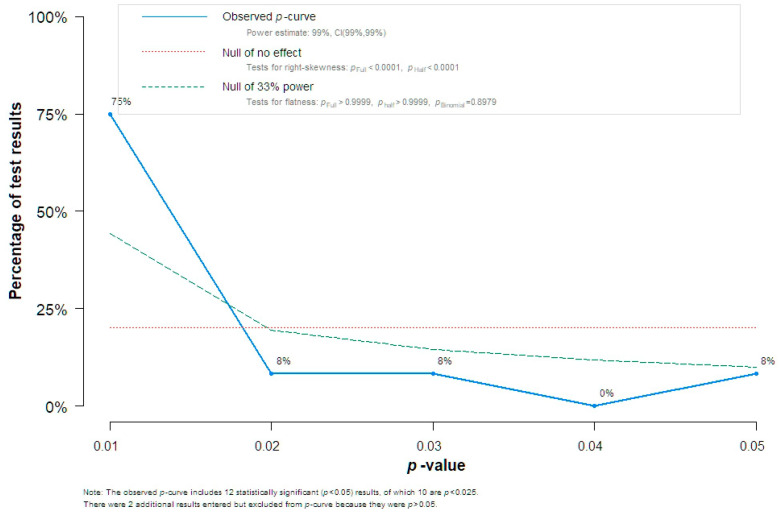
P-curve analysis indicates the absence of p-hacking.

Funnel plot analysis indicated the absence of potential bias, and this was also supported by the Egger’s test analysis (intercept 0.3452, standard error 2.65, *p* = 0.311) (Figure 5. Given this discrepancy in the findings, we chose to perform the Trim n Fill analysis and observed that, with the addition of five hypothetical studies that would provide adequate symmetry to the funnel plot, differences in the summary estimate were non-significant (MD 0.74 ng/mL, 95% CI −7.50, 8.98, outcome based on 18 studies). A P-curve analysis of individual study effect estimates indicated the presence of evidential value, thus denoting the absence of p-hacking (Figure 6). 

Meta-regression analysis indicated that the timing of sample collection (in terms of gestational age) did not influence the summary effect estimate of differences in serum leptin values (outcome based on the 14 studies included in the primary analysis *p*-value = 0.271). The trial sequential analysis revealed that only 25% of the required sample was present to provide adequate power to the meta-analysis (1228 cases of required 5227). 

**Figure 7:**
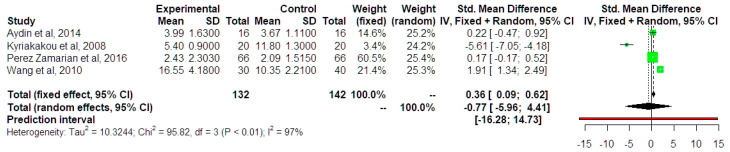
Standardized mean difference of adiponectin between growth-restricted and appropriate-for-gestational-age newborns [13,15,17,18]. Forest plot analysis: Vertical line = “no difference” point between the two groups. Green squares = mean differences of individual studies; Diamond = pooled mean differences and 95% CI for all studies; Horizontal black lines = 95% CI; Horizontal red line = prediction intervals. Abbreviations: SD: Standard Deviation, CI: Confidence Intervals.

The meta-analysis of differences in adiponectin values was based on four studies and revealed the absence of significant differences in standardized mean values (SMD −0.77 ng/mL, 95% CI −5.96, 4.41) (Figure 7). Statistical heterogeneity was particularly high (I^2^ index = 97%). Due to the relatively small amount of included data, sensitivity analysis was not performed, other than the estimation of the summary effect size with the fixed effects model, which revealed that in the absence of significant heterogeneity, the results could indicate a minor increase in adiponectin in IUGR fetuses. Prediction intervals were extremely wide indicating a potentially underpowered effect that would prevent an accurate analysis. The trial sequential analysis indicated that only 0.35% of the required sample size was used; hence, the actual power of the meta-analysis was extremely low to permit accurate determination of the differences in adiponectin levels among the two groups. 

**Figure 8:**
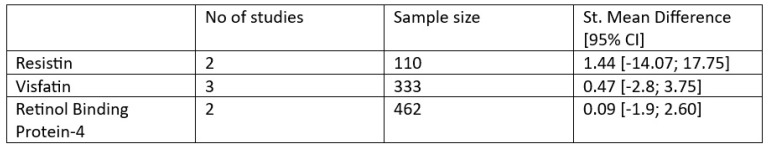
Meta-analysis of the levels of resistin, visfatin, and retinol binding protein-4 between growth-restricted and appropriate-for-gestational-age newborns.

Data related to differences in visfatin, resistin, and RBP4 levels were extremely limited, and the meta-analysis that was performed did not reveal significant differences between IUGR fetuses and controls (Figure 8).

**Figure 9:**
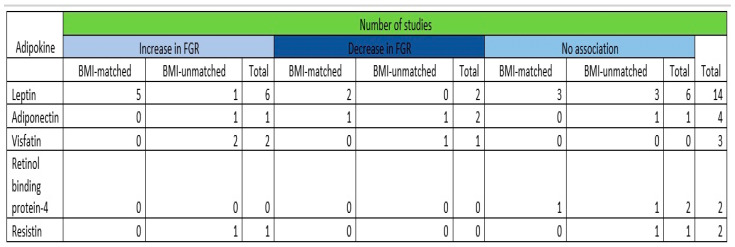
Summary of the qualitative synthesis outcomes, BMI: body mass index, FGR: fetal growth restriction.

A summary of the qualitative synthesis outcomes of the adipokines investigated is depicted in Figure 9. 

## 4. Discussion

### 4.1. Main Findings

The main objective of our study was to accumulate current evidence on the potential association between fetal growth restriction and the levels of the five most studied adipokines (leptin, adiponectin, resistin, visfatin, and retinol binding protein-4). Our meta-analysis revealed that leptin and adiponectin levels are not correlated with growth-restricted fetuses with the results being restricted by the limited data included in the available studies of the literature. In terms of visfantin, resistin, and RBP4 levels, data were extremely limited, and the meta-analysis that was performed did not manage to reveal any statistically significant alterations in growth-restricted fetuses compared to controls.

### 4.2. Comparison with Existing Literature

#### 4.2.1. Leptin and Fetal Growth Restriction

The hormone leptin, which is synthesized by placental tissues, as well as maternal and fetal adipocytes, plays a crucial role in the process of fetal growth throughout an uncomplicated pregnancy [29]. Leptin has a vital role in the development and functioning of the placenta; it is responsible for regulating the formation of the blastocyst and has a crucial function in the processes of implantation and placentation. Leptin induces the production of human chorionic gonadotrophin in trophoblast cells and also influences the proliferation, protein synthesis, invasion, and apoptosis of placental cells [30,31].

Maternal serum leptin concentrations exhibit a normal upregulation in pregnant women [32]. The concentration of serum leptin is significantly elevated in pregnant women compared to women who are not pregnant, with levels reaching two to three times higher [33]. This increase in serum leptin concentration is observed over the gestational period, specifically peaking at approximately 28 to 32 weeks. Following the process of childbirth, hormone levels exhibit a swift decline, returning to their pre-pregnancy levels [34]. The maternal circulating leptin levels experience a notable rise during pregnancy due to an augmented production of leptin from adipose tissue, which is associated with weight gain and fat accumulation in the mother starting from the end of the second trimester. Additionally, the placenta also contributes to the production of leptin as early as the first trimester. This placental production accounts for approximately 15% of the overall concentration of leptin in the mother’s serum.

The studies published so far have demonstrated both higher [9,10,11] and lower [12,13] levels, as well as no association [14,15] between maternal serum leptin levels and fetal growth restriction. Most of the available data suggest that the mean levels of leptin appear to be elevated in growth-restricted fetuses compared to appropriately grown controls, a difference that becomes further aggregated when growth restriction is accompanied by the development of preeclampsia [35]. Contradictory to the above data, there are studies that have demonstrated lower maternal serum leptin levels between growth-restricted cases and uncomplicated controls. However, the findings of the current published bibliography alongside the results of our meta-analysis suggest that leptin may indeed have a role in the pathophysiology of several adverse events of the pregnancy, and it has been proposed that the comparison of leptin concentrations against the reference values that have been published [36] might be a useful prediction asset of such complications [14]. The studies conclude that both the up- and down-regulation of serum leptin is originating from the deterioration of the placenta, with a number of published data revealing the critical effect of those alterations in preterm newborns without being limited in the antenatal period but also influencing the growth trajectories of the affected neonates [14]. 

#### 4.2.2. Adiponectin and Fetal Growth Restriction

Adiponectin is a hormone primarily secreted by adipose tissue cells and plays a significant role in the regulation of metabolism as well as in the regulation of insulin sensitivity. Additionally, it promotes anti-inflammatory and anti-atherogenic activities and vasodilation, while also playing a significant role in the pathogenesis of metabolic syndrome [37,38,39,40]. Adiponectin levels have shown a negative correlation with body mass index and may decrease in conditions related to obesity and insulin resistance [41,42].

During pregnancy, adipose tissue is the main source of maternal adiponectin production. The involvement of the placenta in this process, as well as the degree to which adiponectin is expressed by the placenta, remains unclear [16,43,44,45]. Adiponectin levels in pregnant women are reduced, particularly in the third trimester of pregnancy, when maternal insulin resistance is at its highest [44,46,47].

Various studies have suggested the possible involvement of adiponectin in fetus development [48,49]. The involvement of maternal adiponectin in fetus development is primarily attributed to its role in metabolic adaptation during pregnancy. Specifically, unregulated adiponectin expression inhibits the insulin signaling pathway and the transport of amino acids to the placenta, which, in turn, affects the transfer of nutrients to the embryo and subsequently leads to abnormal fetus development and growth [50].

The published studies have demonstrated conflicting results, regarding the correlation between maternal serum adiponectin levels and fetal growth restriction. In particular, Kyriakakou et al. and Visentin et al. report statistically significant lower levels of circulating adiponectin in IUGR mothers than in uncomplicated control groups [10,17], while Wang et al. presented results that indicate a statistically significant positive correlation between increased maternal adiponectin levels and FGR. The conflicting findings may be due to the fact that adiponectin gene expression is a complex multifactorial process and that adiponectin maternal levels during pregnancy are related to various environmental and genetic factors such as insulin resistance, obesity, and ethnicity [51]. The gestational age that adiponectin levels were examined, the population heterogeneity, and the study design are additional causes of conflicting findings. 

Due to the very small sample and the heterogeneity of published data, our meta-analysis was unable to clarify the correlation between the presence of FGR babies and circulating maternal adiponectin levels.

#### 4.2.3. Visfatin and Fetal Growth Restriction

Visfatin is a protein secreted by adipose tissue and has a role in insulin sensitivity regulation, energy metabolism, and inflammatory response. While its exact function is still the subject of ongoing research, there is some evidence to suggest that visfatin may play a role in fetal development, due to the fact that visfatin production is not limited to adipose tissue but is also expressed in placenta, in the myometrium, and in fetal membranes [6,52,53,54,55,56]. In normal pregnancies, higher maternal serum visfatin levels have been observed [57,58,59].

Studies published so far indicate that there is an association between maternal visfatin levels and fetal growth restriction, but this correlation is not yet conclusive. In particular, Malamitsi-Puchner et al. investigated the levels of maternal visfatin in 20 mothers of full-term infants with IUGR and compared them with those of 20 mothers who gave birth to AGA infants. Their research concluded that the values were significantly higher in mothers who gave birth to IUGR infants. On the same hand, Mazaki-Tovi et al. compared the levels of maternal serum visfatin in 158 normal pregnancies with the levels of maternal serum visfatin levels in 55 women who delivered SGA infants and concluded that maternal visfatin levels were higher in pregnancies with SGA infants than in normal pregnancies. Pekal et al. measured maternal visfatin concentration in 40 women with SGA neonates and compared it with visfatin concentration in 40 women with AGA neonates, and in contrast with the above, came to the conclusion that visfatin concentration levels were significantly lower in mothers with SGA infants than in mothers with AGA infants [19,20,60].

Ιn accordance with the above, it is understood that there is no generally accepted conclusion regarding the relationship and correlation between maternal visfatin levels and the birth of neonates with FGR. The existing data made it impossible to carry out a meta-analysis which can safely clarify the exact correlation between maternal visfatin concentration and the birth of a growth-restricted infant.

#### 4.2.4. Resistin and Fetal Growth Restriction

Resistin is an adipokine that has been studied extensively since its discovery more than 20 years ago; it is considered to be produced by the placenta and is considered to be a regulator of insulin resistance in pregnant women [61]. Furthermore, studies have produced inconsistent results as to whether resistin levels impact fetal growth and to what extent, if there is an association.

An earlier analysis by Struwe et al., which included 16 small for gestational age (SGA) and 24 appropriate for gestational age (AGA) children, showed no significant difference between the two groups [62]. An analysis which compared SGA children at baseline with controls born appropriate size for age showed that resistin levels were lower for SGA children [63]. On the other hand, another study which included 45 SGA children showed higher levels of resistin, and it was independently correlated with low birth weight [64]. Recently, a study of 35 SGA children and 25 AGA children failed to show any difference in resistin levels between these two groups [65].

#### 4.2.5. Retinol Binding Protein-4 and Fetal Growth Restriction

Retinol binding protein 4 (RBP 4) is an adipokine produced in the liver and has been implicated in obesity-induced insulin resistance and inflammation. Currently, there is intense research regarding the association of RBP 4 levels in pregnant women with preeclampsia.

A retrospective study by Vaisbuch et al. included patients in four different categories: normal pregnancy, preeclampsia, small for gestational age (SGA), and fetal death [21]. For all these patients, they calculated RBP 4 levels. They found that RBP 4 levels correlated positively with pregnant women that had preeclampsia compared to normal pregnancy. In addition, higher RBP 4 concentration was also observed with preterm preeclampsia (<37 weeks) compared to women with term preeclampsia. Furthermore, no association was observed in SGA or fetal death pregnancies.

In another, which included 480 singleton pregnancies (240 with normal outcomes, 60 with preeclampsia, 60 with gestational diabetes mellitus, 60 with SGA, and 60 with large for gestational age), the authors measured RBP 4 serum levels between 11 and 13 weeks [22]. The authors concluded that RBP 4 levels did not correlate with fetal growth alterations. In our study, the scarcity of existing data made it impossible to carry out a meta-analysis, and more data seem to be required for conclusions to be drawn. 

### 4.3. Strengths and Limitations

To the best of our knowledge, this is the first meta-analysis to assess the levels of leptin, adiponectin, visfatin, resistin, and RBP4 with fetal growth restriction. The main strength of the review lies on the fact that an extensive search strategy was applied in an effort to include all the available literature of the issue under review, with our review comprising a sample size based on a total of 20 studies and 1850 patients. Furthermore, the majority of the included studies were matched for BMI, a fact that minimizes the risk for the reported results to be influenced by the presence of the aforementioned confounding factor that is known to affect the levels of the adipokines under examination. The credibility of evidence was also evaluated, proposing a high quality of evidence. 

We acknowledge that the present meta-analysis has dealt with several limitations. Several parameters contribute to this finding. For instance, the methodological heterogeneity that was noted in these studies may result in significant selection bias that may prohibit clear conclusions. To be more precise, different criteria to define fetal growth restriction have been applied among the different studies, since the included studies span over a period of 20 years with the diagnostic criteria of fetal growth restriction having been exposed to several changes during this study period. Furthermore, the available data were quite limited for solid conclusions to be able to be extracted in terms of every adipokine under examination. More specifically, leptin, which is the adipokine most studied, was examined in a total of 357 fetal growth-restricted fetuses and 724 appropriately grown ones, a study population that restricted our review from drawing firm conclusions. Lastly, due to the aggregated nature of included data, potential sources of bias, including gender or associated medical conditions, could not be controlled for, and that seems to be an issue that requires further research in the future.

## 5. Conclusions

The existing evidence suggests that fetal growth restriction is not associated with the levels of leptin, adiponectin, visfatin, resistin, and RBP4, but the available data and published studies are currently either limited or conflicting. Therefore, it is imperative to conduct further extensive prospective studies to provide a comprehensive understanding of the significance of already established and emerging adipokines, in order to validate or refute the findings of our review and, if so, to provide universally applicable threshold values for clinical implementation.

## Data Availability

Publicly available datasets were analyzed in this study.

## Supplementary Materials

The following supporting information can be downloaded at: https://www.mdpi.com/article/10.3390/jcm13061667/s1. Table S1: The methodological characteristics of the included studies that investigated leptin; Table S2: Demographic characteristics of the included patients whose leptin levels were assessed; Table S3: The methodological characteristics of the included studies that investigated adiponectin; Table S4: Demographic characteristics of the included patients whose adiponectin levels were assessed.
