# Supplementary material for: Maternal Blood Adipokines and Their Association with Fetal Growth: A Meta-Analysis of the Current Literature
Source: J Clin Med. 2024 Mar 14;13(6):1667. doi: 10.3390/jcm13061667 (PMC10971104; doi:10.3390/jcm13061667)
Supplement: Supplementary file 1 [file jcm-13-01667-s001.zip › Table S1.pdf]

**Methodological characteristics of included studies (maternal serum leptin)**			
Year; author	Study type	Inclusion criteria	Exclusion criteria
Nezar et al, 2008 [14]	Prospective cohort	• Singleton pregnancies<br>• FGR: EFW$<10^{TH}$ centile	• Multiple pregnancies<br>• Evidence of chromosomal abnormalities or fetal anatomic defects<br>• Consent not given
Stefaniak et al, 2022 [16]	Prospective cohort	• Singleton pregnancies<br>• FGR: AC$<5^{TH}$ centile + BW$<10^{th}$ centile	• Multiple pregnancies<br>• Consent not given
Visentin et al, 2014 [15]	Retrospective cohort	• Singleton pregnancies<br>• FGR: EFW $<10^{TH}$ centile + UA Pulsatility Index > 2 SD	• Multiple pregnancies<br>• Evidence of chromosomal abnormalities or fetal anatomic defects<br>• Consent not given<br>• Pregnancies complicated by maternal history of cardiovascular disease or endocrine disorder<br>• Clinical chorioamnionitis<br>• Consumption of alcohol, smoke, nicotine, or any medication such as ritodrine and corticosteroids (except for fetal lung maturation)<br>• Amniotic fluid disorders<br>• Placental abnormalities
Zareaan et al, 2017 [18]	Cross – sectional study	• maternal age <40 yr<br>• singleton pregnancy<br>• gestational age >36 weeks<br>• non-smoking and substance abuse or teratogenic drug<br>• body mass index (BMI) between 20-27 Kg/m^{2}P P	• Multiple pregnancies<br>• Inability to determine the level of leptin<br>• Withdrawal of participants<br>• Pregnancy complications<br>• Systemic problems including: kidney disease, anemia, history of diabetes before pregnancy, heart disease<br>• FGR in previous pregnancy<br>• Pregnancy with assisted reproductive techniques (ART)<br>• Congenital malformations
Aydin et al, 2015 [20]	Prospective case control	• IUGR: BW or length $<10^{th}$ percentile with pathological fetal growth restriction<br>• Maternal age 18-40<br>• Singleton pregnancy<br>• Gestational age 36-40<br>• IUGR diagnosed in second or third trimester	• BW> 10th percentile<br>• Multiple pregnancies<br>• Systemic illness (gestational diabetes, preeclampsia, eclampsia, chronic hypertension, vascular diseases, thrombophilia)<br>• Infectious diseases<br>• Uterine malformation<br>• Smoking<br>• Fetuses with congenital malformation or chromosomal anomaly<br>• Consent not given
Catov et al, 2007 [17]	Nested case control study	• SGA: <10th percentile<br>• Nulliparous<br>• Normotensive<br>• Maternal age 14-44<br>• <20 weeks' gestation when enrolled<br>•	• Preeclampsia<br>• Transient hypertension<br>• Multiple gestation<br>• Gestational diabetes<br>• Chronic conditions<br>• Positive toxicology screen<br>• Consent not given
Ferrero et al, 2014 [61]	Prospective study	• FGR: <10th percentile and normal Doppler values	• Consent not given<br>• Use of drugs that may interfere with fetal growth or biochemical markers<br>• Genetic alterations of growth potential<br>• Gestational diabetes<br>• Multiple pregnancy<br>• Hypertension or preeclampsia<br>• Pregnancy not controlled at the hospital<br>• Delivery attended in other hospital
Garofoli et al,2022 [19]	Prospective longitudinal observational study	• IUGR: flussimetric findings during ultrasound imaging and did not reach their genetically determined potential growth<br>• Breastfeeding until the end of the third month of life	• Infants with congenital malformations<br>• Infants with infection<br>• Genetic syndromes<br>• Formula fed<br>• Infants large for gestational age<br>• Consent not given

Jenkins et al, 2007 [60]	Case control study	• SGA: BW<10th centile<br>• Normal controls: no clinical signs of hypertension, diabetes or infection	• Consent not given
Kyriakakou et al, 2008 [38]	Prospective cohort	• IUGR: BW<3rd centile<br>• Full term singleton infants	• Consent not given
Laivuori et al, 2006 [62]	Cross sectional study	• IUGR<br>• Written informed consent<br>• Controls: uncomplicated pregnancies	• Preexisting diabetes<br>• Consent not given
Lepercq et al, 2002 [63]	Cross sectional study	• Normotensive IUGR : BW<10th percentile<br>• Controls: AGA preterm infants<br>Informed consent	• Diabetes<br>• Obesity<br>• Consent not given
Mise et al, 2007 [64]	Retrospective cohort	• Group A preeclampsia ± SGA<br>• Group B non preeclampsia ±SGA<br>• SGA: BW<10th percentile	• Consent not given

Table S1. The methodological characteristics of the included studies that investigated leptin. Abbreviations: **FGR**: Fetal Growth Restriction, **IUGR**: Intrauterine Growth Restriction, **EFW**: Estimated Fetal Weight, **SGA**: Small for Gestational Age, **BW**: Birth Weight, **SD**: Standard Deviation.
