# Supplementary material for: Maternal Blood Adipokines and Their Association with Fetal Growth: A Meta-Analysis of the Current Literature
Source: J Clin Med. 2024 Mar 14;13(6):1667. doi: 10.3390/jcm13061667 (PMC10971104; doi:10.3390/jcm13061667)
Supplement: Supplementary file 1 [file jcm-13-01667-s001.zip › Table S2.pdf]

Patient characteristics (Cases vs. **Controls**)

Year; author	N	Age	Pre-pregnancy BMI	BMI at birth	Race (White/Black/Other)	DM	Nulliparous	Smoking	PIH	PET	Cesarean section	Gestational age at delivery	Birthweight	APGAR Score	Umbilical cord pH
Nezar et al, 2008	20/**15**	27.1/**28.4**	28.7/**29.4**	n/a	n/a	n/a	n/a	n/a	n/a	n/a	8 / **11**	37.4 / **38.5**	2250 / **3250**	9.1 / **10**	n/a
Stefaniak et al, 2022	25/**194**	31/**30**	n/a	25.7 / **28.1**	n/a	n/a	n/a	n/a	n/a	n/a	n/a	37 / **38.9**	2440 / **3454**	n/a	n/a
Visentin et al, 2014	37/**70**	32.70 /**32.00**	23.80 /**22.1**	n/a	n/a	n/a	25 / **37**	n/a	n/a	n/a	23 / **32**	36.75 / **38.57**	2131.67 /**3178.17**	n/a	n/a
Zareaan et al, 2017	40/40	27.28 /**28.05**	23.04 / **23.18**	n/a	n/a	n/a	n/a	0 / **0**	n/a	n/a	n/a	37.10 /**37.35**	2321.25 / **3219.5**	n/a	n/a
Aydin et al, 2015	16/**16**	29.6 / **30.7**	22.9 / **22.9**	27.7 / **27.9**	16/0/0 /**16/0/0**	0/**0**	n/a	0/**0**	0/**0**	0/**0**	n/a	37.5 / **38.0**	2384 / **3218**	n/a	n/a
Catov et al, 2007	28/**77**	22.9 / **21.9**	n/a	24.6 / **23.8**	18white+other/10 black / **53white+other/24black**	0 / **0**	28/**12**	0/**0**	0/**0**	0/**0**	n/a	39.6 / **39.7**	2489 / **3342**	n/a	n/a
Ferrero et al, 2014	46/**81**	29.9 / **31.8**	n/a	22.3 / **23.4**	43 Spanish nationality / **79 Spanish nationality**	0/**0**	n/a	18/**16**	0/**0**	0/**0**	n/a	37.3/**39.4**	2127/**3270**	n/a	Arterial pH 7.23 / **7.26** Venous pH 7.29/**7.31**
Garofoli et al, 2022	13/**16**	32/**31**	23/**23.5**	26/**28**	n/a	n/a	n/a	n/a	n/a	n/a	10 /**1**	36.6 / **40.5**	1770.0 /3242.5	9/10	n/a
Jenkins et al, 2007	A)27/**30**<br>B)30/**30**	A)20.9/**20.9**<br>B)25.9/**28.9**	A)22.7/**23.5**<br>B)23.5/**23.8**	A)n/a<br>B) n/a	A)27 black/0white +other/**30 black**<br>B) 30 white/0black +other/ **30 black**	n/a	n/a	n/a	n/a	n/a	n/a	A)39.6/ **39**<br>B)38.8/**39.4**	A)2501/**3073**<br>B)2348/**3560**	n/a	n/a
Kyriakakou et al, 2008	20/**20**	30.8/**31.2**	n/a	22.9/21.4	n/a	10/**9**	17/**9**	5/**0**	n/a	n/a	12/**6**	265.6(days)/**272(days)**	2379/**3160**	≥8/ **≥8**	n/a
Laivuori et al, 2006	20/**18**	21.7/**21.2**	22.6/**22.7**	n/a	11/9/0 / **0/18/0**	0/**0**	17/**18**	14/**8**	0/**0**	0/**0**	2/**2**	39.7/**39.8**	n/a	n/a	n/a
Lepercq et al, 2002	13/**9**	31.4/**30.7**	n/a	21.7/24.3	n/a	0/**0**	n/a	n/a	0/**0**	0/**0**	n/a	35.2/**33.5**	1711/**2351**	n/a	Arterial 7.33/**7.27**
Mise et al, 2007	A)15/**25**<br>B)11/**87**	A)30.7/**30.5**<br>B)32.1/**28.2**	n/a	n/a	n/a	n/a	n/a	n/a	A)0/**0**<br>B)n/a	A)15/**25**<br>B)0/**0**	A)n/a<br>B)n/a	n/a	n/a	n/a	n/a

Table S2. Demographic characteristics of the included patients whose leptin levels were assessed. Abbreviations: **BMI**: Body Mass Index, **DM**: Diabetes Mellitus, **PIH**: Pregnancy Induced Hypertension, **PET**: Preeclampsia, **n/a**: not applicable
