# Supplementary material for: Maternal Blood Adipokines and Their Association with Fetal Growth: A Meta-Analysis of the Current Literature
Source: J Clin Med. 2024 Mar 14;13(6):1667. doi: 10.3390/jcm13061667 (PMC10971104; doi:10.3390/jcm13061667)
Supplement: Supplementary file 1 [file jcm-13-01667-s001.zip › Table S3.pdf]

Methodological characteristics of included studies (maternal serum adiponectin)			
Year; author	Study type	Inclusion criteria	Exclusion criteria
Visentin et al, 2014 [15]	Retrospective cohort	• Singleton pregnancies<br>• FGR: EFW <10TH centile + UA Pulsatility Index > 2 SD	• Multiple pregnancies<br>• Evidence of chromosomal abnormalities or fetal anatomic defects<br>• Consent not given<br>• Pregnancies complicated by maternal history of cardiovascular disease or endocrine disorder<br>• Clinical chorioamnionitis<br>• Consumption of alcohol, smoke, nicotine, or any medication such as ritodrine and corticosteroids (except for fetal lung maturation)<br>• Amniotic fluid disorders<br>• Placental abnormalities
Aydin et al, 2015 [20]	Prospective case control	• IUGR: BW or length <10th percentile with pathological fetal growth restriction<br>• Maternal age 18-40<br>• Singleton pregnancy<br>• Gestational age 36-40<br>• IUGR diagnosed in second or third trimester<br>•	• BW> 10th percentile<br>• Multiple pregnancies<br>• Systemic illness (gestational diabetes, preeclampsia, eclampsia, chronic hypertension, vascular diseases, thrombophilia)<br>• Infectious diseases<br>• Uterine malformation<br>• Smoking<br>• Fetuses with congenital malformation or chromosomal anomaly<br>• Consent not given
Kyriakakou et al, 2008 [38]	Prospective cohort	• IUGR: BW<3rd centile<br>• Full term singleton infants	• Consent not given
Wang et al, 2010 [39]	Prospective cohort	• Singleton pregnancies<br>• No obstetrical, maternal, or fetal complications<br>• Normal plasma glucose concentration in the first trimester<br>• Normal OGTT in the third trimester and<br>• Full term 37–42 weeks	• Unreliable gestational age,<br>• pregnancies after ovulation induction or in vitro fertilisation<br>• Pregnancy- induced hypertension,<br>• Pre-gestational or gestational diabetes mellitus<br>• previous chronic diseases (e.g. epilepsy, renal insufficiency and heart disease)<br>• Medical treatment, especially glucocorticoids.

Table S3. The methodological characteristics of the included studies that investigated adiponectin. Abbreviations: **FGR**: Fetal Growth Restriction, **IUGR**: Intrauterine Growth Restriction, **EFW**: Estimated Fetal Weight, **BW**: Birth Weight, **UA**: Umbilical Artery, **SD**: Standard , **OGTT**: Oral Glucose Tolerance test
