# Supplementary material for: Maternal Blood Adipokines and Their Association with Fetal Growth: A Meta-Analysis of the Current Literature
Source: J Clin Med. 2024 Mar 14;13(6):1667. doi: 10.3390/jcm13061667 (PMC10971104; doi:10.3390/jcm13061667)
Supplement: Supplementary file 1 [file jcm-13-01667-s001.zip › Table S4.pdf]

Patient characteristics (Cases vs **Controls**)															
Year; author	N	Age	Pre-pregnancy BMI	BMI at birth	Race (White/Black/Other)	DM	Nulliparous	Smoking	PIH	PET	Cesarean section	Gestational age at delivery	Birthweight	APGAR Score	Umbilical cord pH
Visentin et al, 2014	37/**70**	32.70 /**32.00**	23.80 /**22.1**	n/a	n/a	n/a	25 / **37**	n/a	n/a	n/a	23 / **32**	36.75 / **38.57**	2131.67 /**3178.17**	n/a	n/a
Aydin et al, 2015	16/**16**	29.6 / **30.7**	22.9 / **22.9**	27.7 / **27.9**	16/0/0 /**16/0/0**	0/**0**	n/a	0/**0**	0/**0**	0/**0**	n/a	37.5 / **38.0**	2384 / **3218**	n/a	n/a
Kyriakakou et al, 2008	20/**20**	30.8/**31.2**	n/a	22.9/21.4	n/a	10/**9**	17/**9**	5/**0**	n/a	n/a	12/**6**	265.6(days)/**272(days)**	2379/**3160**	≥8/ **≥8**	n/a
Wang et al, 2010	30/**40**	27.6 /**26**	n/a / **23.5**	n/a	n/a	n/a	n/a	n/a	n/a	n/a	n/a	275.8 (days)/**276.9(days)**	2300 /**3700**	n/a	n/a

Table S4. Demographic characteristics of the included patients whose adiponectin levels were assessed. Abbreviations: **BMI**: Body Mass Index, **DM**: Diabetes Mellitus, **PIH**: Pregnancy Induced Hypertension, **PET**: Preeclampsia, **n/a**: not applicable
